# Sex discrepancies in pathophysiology, presentation, treatment, and outcomes of severe aortic stenosis

**DOI:** 10.3389/fcvm.2023.1256970

**Published:** 2023-08-15

**Authors:** Julia Stehli, Sarah Zaman, Barbara E. Stähli

**Affiliations:** ^1^ Department of Cardiology, University Hospital Zurich, Zurich, Switzerland; ^2^ Westmead Applied Research Centre, Faculty of Medicine and Health, University of Sydney, Sydney, NSW, Australia; ^3^ Department of Cardiology, Westmead Hospital, Sydney, NSW, Australia; ^4^ Faculty of Medicine, University of Zurich, Zurich, Switzerland

**Keywords:** female, sex, aortic stenosis, transcatheter aortic valve implantation (TAVI), surgical aortic valve replacement (SAVR)

## Abstract

This review gives an overview of sex-based differences in aortic valve stenosis, spanning from pathophysiological mechanisms and disease progression, clinical presentation, presence of comorbidities, and diagnostic assessment, to treatment and outcomes. In particular, sex-related differences in the degree of aortic valve calcification, the response of the left ventricle to pressure overload, as well as in the referral to procedures, with women being less frequently referred for surgical aortic valve replacement and experiencing longer waiting times for transcatheter procedures, will be discussed. Sex-related differences are also particularly evident in outcomes of patients with severe aortic stenosis undergoing surgical or transcatheter procedures. The apparent sex paradox seen in women undergoing transcatheter aortic valve implantation refers to the phenomenon of women experiencing higher rates of short-term mortality and bleeding events, but demonstrating improved long-term survival as compared to men. Women who undergo surgical aortic valve replacement have generally worse outcomes as compared to men, which is reflected by the inclusion of female sex in surgical risk calculation scores. Hence, a thorough understanding of sex-related differences in aortic valve stenosis is important to provide optimal and personalized patient care.

## Introduction

Aortic stenosis (AS) is highly prevalent and known to increase in prevalence with age (1). In the elderly, severe AS is present in 3.4% of the population and any degree of AS in up to 12.4% (2). Interestingly, sex distribution differs across age subgroups. In younger patients there is a male predominance, primarily due to the prevalence of bicuspid aortic valve (AV) disease (3, 4). However, women represent the majority of patients with severe AS over 80 years of age (2, 5).

### Pathophysiology

The development of AS shares several pathophysiological similarities with atherosclerosis. Endothelial damage sets off a cascade of processes involving lipid accumulation, inflammation, the development of fibrotic alterations, and finally calcification (6). Interestingly, it has been observed that women with similar hemodynamic AS severity show a lower degree of AV calcification than men (7). It is therefore of utmost importance to consider sex-specific threshold values for the definition of severe AS by means of computed tomography (CT) measurement of AV calcification. The [European Society of Cardiology (ESC)] guidelines suggest cutoffs of >3,000 AU in men and >1,600 AU in women as “highly likely” for severe AS. Calcium scoring of >2,000 AU in men and >1,200 AU in women “likely” represent severe AS, whereas values of <1,600 AU in men and <800 AU in women most likely do not represent severe AS (8). This discrepancy in the degree of AV calcification persists even after accounting for the smaller size (body, heart, and aorta) of women, with severe AV calcification relative to the aortic annulus area defined as ≥300 Agatston unit (AU)/cm^2^ in women and ≥500 AU/cm^2^ in men (9). When accounting for the lower CT calcium score at baseline, female sex has been identified as independent predictor of AV calcification progression (10). Notably, in patients undergoing transcatheter aortic valve implantation (TAVI), a significant increase in mortality risk was observed with higher AV calcium scores among women. Every 500 AU increase in AV calcium was associated with a 7% increase in mortality risk in women, while no significant association was observed in men (11).

The response of the left ventricle to pressure overload may also demonstrate sex-related differences, however, results are conflicting. In an analysis performed in patients waiting for surgical aortic valve replacement (SAVR), women more frequently exhibited left ventricular concentric remodeling as compared to men which more often displayed eccentric left ventricular remodeling (12). However, in a study enrolling elderly patients scheduled for TAVI, no differences between sexes were observed in left ventricular remodeling patterns as assessed by CT scans (13), although smaller left ventricular volumes and mass were observed for women, even after indexing to body surface area (13).

Another difference is the development of a more diffuse fibrosis pattern within the myocardium in women as compared to the higher level of focal fibrosis in men (14). This could be the reason that women exhibit more often reduced left ventricular compliance, higher left ventricular filling pressures, increased left atrial volume indexes, and consequentially more advanced left ventricular diastolic dysfunction and higher rates of heart failure with preserved left ventricular ejection fraction (12, 15). This remodeling pattern may promote the development of paradoxical low-flow AS in women and emphasizes the importance of considering sex-specific factors in the assessment and management of patients with AS and heart failure. Indeed, sex specific cut-off values for indexed stroke volume have been proposed (40 ml/m^2^ for men and 32 ml/m^2^ for women) (16). Alongside increased myocardial fibrosis, differences in ventricular remodeling may also be influenced by the higher prevalence of hypertension in women and possible interactions with sex hormones (17).

### Symptoms and presentation

Women with severe AS tend to receive their diagnosis at later ages as compared to men (15), and they are less likely to have concomitant coronary artery disease, peripheral arterial disease, and amyloid cardiomyopathy (17, 18). When presenting with severe AS, even when the severity of AS is similar, women experience a greater symptom burden (15, 19). This includes a higher incidence of exertional dizziness and more pronounced dyspnea. Women also tend to have an increased level of frailty at the time of presentation, which is a known risk factor for worse outcomes in these patients (19–21).

### Prognosis and treatment

Even the presence of mild stenotic AV changes is associated with a 50% higher risk of myocardial infarction and cardiovascular death (6). Once severe AS has developed and patients become symptomatic, rates of mortality rise to more than 30% per year if AS is left untreated (22). Similar mortality rates of women and men with untreated severe AS have been observed (22, 23).

There are data pointing towards an undertreatment of patients with severe AS. In a large US cohort of 43,000 patients diagnosed with severe AS between 2008 and 2016, only 28% of patients underwent SAVR or TAVI within one year of diagnosis (22). Even after adjusting for clinical characteristics, socioeconomic status, and access to healthcare, women were 20% less likely than men to undergo AV replacement, including both SAVR and TAVI (15). Further, women appear to be referred at a later stage of the disease compared to men (15).

### Transcatheter aortic valve implantation

Current guidelines recommend TAVI as a Class I indication for patients over 65 year years of age (American College of Cardiology (ACC)/American Heart Association (AHA)) or over 75 years of age ESC who are at high or prohibitive risk for SAVR and suitable candidates for transfemoral TAVI (8, 24). Based on these guidelines, approximately 290,000 elderly individuals with severe AS may qualify as candidates for TAVI and yearly around 27,000 become eligible for TAVI (2). It needs to be taken into account that although females represent half of the patients included in registries (25, 26), women have consistently been underrepresented in large TAVI trials, with only 33%–47% of participants being females (27–29).

Among the women who underwent any kind of AV procedure, a higher proportion received TAVI as compared to men (22, 25). Women undergoing TAVI were mostly older and had higher levels of frailty than men, but fewer comorbidities (21). Further, longer waiting times to undergo TAVI, including longer work-up and procedural waiting times, were observed in women, even after adjustment for comorbidities and age (19). The lower referral rates of women for SAVR as well as the longer waiting times for TAVI may at least in part represent health care system-related delays. However, whilst it is possible that the risk of women with severe AS may be underestimated, as it has been documented in coronary artery disease (30, 31), it is also possible that women themselves contribute to prolonged waiting times due to potential misinterpretation of symptoms and their roles as caregivers in the society (32).

In terms of procedural outcomes, no differences in procedural success were observed between men and women undergoing TAVI (26). However, it is worth noting that female sex is associated with a higher incidence of major bleeding and major vascular access site complications (25, 33, 34). In a recently published *post hoc* analysis of the Antiplatelet Therapy for Patients Undergoing Transcatheter Aortic Valve Implantation (POPular TAVI) trial, the influence of sex on bleeding and ischemic complications following TAVI was examined, taking into account the specific antiplatelet and anticoagulation regimens used (35). This analysis showed that the overall incidence of bleeding events did not differ between women and men, but women exhibited a higher occurrence of major or life-threatening bleedings as compared to men. An interesting observation of this study was the differential effect of antithrombotic treatment on bleeding events in women and men. Specifically, women who received aspirin both before and after TAVI had a higher incidence of major or life-threatening bleeding as compared to men (35). Several mechanisms have been suggested to account for post-TAVI bleeding, encompassing intrinsic bleeding abnormalities that go beyond the platelet system (36). These mechanisms may differ from those observed in patients with coronary artery disease who undergo percutaneous coronary intervention, where bleeding issues are also more prevalent in women (37). Since coexistence of epicardial coronary artery disease among patients with AS is common and both pathologies lead to similar symptoms, decisions making regarding treatment of either AS or coronary artery disease or both remains a challenge. This is particularly true since treatment for coronary artery disease will require dual antiplatelet therapy, increasing the risk of bleeding in women undergoing TAVI (35). Further clarification on which patients are more susceptible to bleeding after TAVI might arise from subanalyses of data derived from the Global Study Comparing a Rivaroxaban-based Antithrombotic Strategy to an Antiplatelet-based Strategy after Transcatheter Aortic Valve Replacement to Optimize Clinical Outcomes (GALILEO) (38).

Following TAVI, more women than men were discharged to rehabilitation facilities, likely due to a higher level of frailty observed in women as compared to men (19, 21).

Short-term mortality and rates of readmission following TAVI have decreased over time across both women and men (34). Despite these improvements, women undergoing TAVI continue to have higher rates of in-hospital mortality and 90-day readmission as compared with men (39). The higher bleeding rates could explain at least in part the higher short-term mortality that was observed in women in multiple analyses (26, 33, 34). Despite increased rates of bleeding and vascular access site complications as well as an excess short-term mortality, multiple observational studies and meta-analyses pointed towards better mid- and long-term survival among women who undergo TAVI as compared to men (40). However, whether sex-related differences in long-term survival after TAVI do exist remains to be determined, as recent analyses of large randomized trials in high- and intermediate-risk patients undergoing TAVI with newer generation transcatheter heart valves have revealed no sex-related differences in survival (20). Changing demographics of enrolled patients, the utilization of newer-generation transcatheter heart valves with smaller delivery systems, more accurate valve sizing techniques, as well as increasing operator experience may have substantially impacted on outcomes in women and men after TAVI. Consistently, observational analyses confirmed a decrease in mortality rates following TAVI over time, however, mortality rates decreased to a greater extent in men than in women (60% vs. 50%) (34). The ongoing randomized controlled Randomized researcH in womEn All Comers With Aortic Stenosis (RHEIA) trial, comparing SAVR and TAVI specifically in women, will provide important insights into this topic (41).

Worse outcomes for women undergoing TAVI were observed in specific vulnerable subsets of patients, including those with low-gradient low-ejection fraction AS. In this patient cohort, women undergoing TAVI exhibited significantly higher rates of mortality as compared to men (17, 23). This disparity in long-term survival could be attributed to the distinct and sex-specific left ventricular remodeling pattern induced by pressure overload.

Other subgroups of patients in which women had an increased mortality were very elderly, frail patients as well as those with pulmonary hypertension (21, 42). The longer waiting times for TAVI observed in women were also associated with higher rates of mortality and hospitalizations for heart failure and reduced mobility (19).

A comparable symptomatic benefit after TAVI has been reported for women and men, with similar improvements in quality of life (evaluated by the Kansas City Cardiomyopathy Questionnaire) observed following TAVI, which is an interesting finding, given the increased age and the higher prevalence of frailty as well as reduced mobility observed in women (43).

### Surgical aortic valve replacement

The impact of sex on outcomes following SAVR remains a subject of ongoing debate. Various analyses have reported worse outcomes for women undergoing SAVR, including an excess short-term mortality, an increased need for postoperative blood products, and a longer hospital stay (44, 45). However, women are also older and have more advanced disease at the time of surgery as compared to men, which is likely one of the causes for the increased mortality observed after SAVR in women (22). Other factors that may contribute to these sex-related differences are increased frailty, a higher prevalence of patient prosthesis mismatch due to smaller aortic annular dimensions, a higher incidence of paradoxical low-flow AS, and an increased need for permanent pacemaker implantation in women (23). In some analyses, female sex itself has been identified as independent predictor of mortality and morbidity following SAVR (46). Consequentially, female sex is included in the widely used Society of Thoracic Surgeons (STS) and EuroSCORE surgical risk prediction tools (47, 48).

**CENTRAL ILLUSTRATION:**
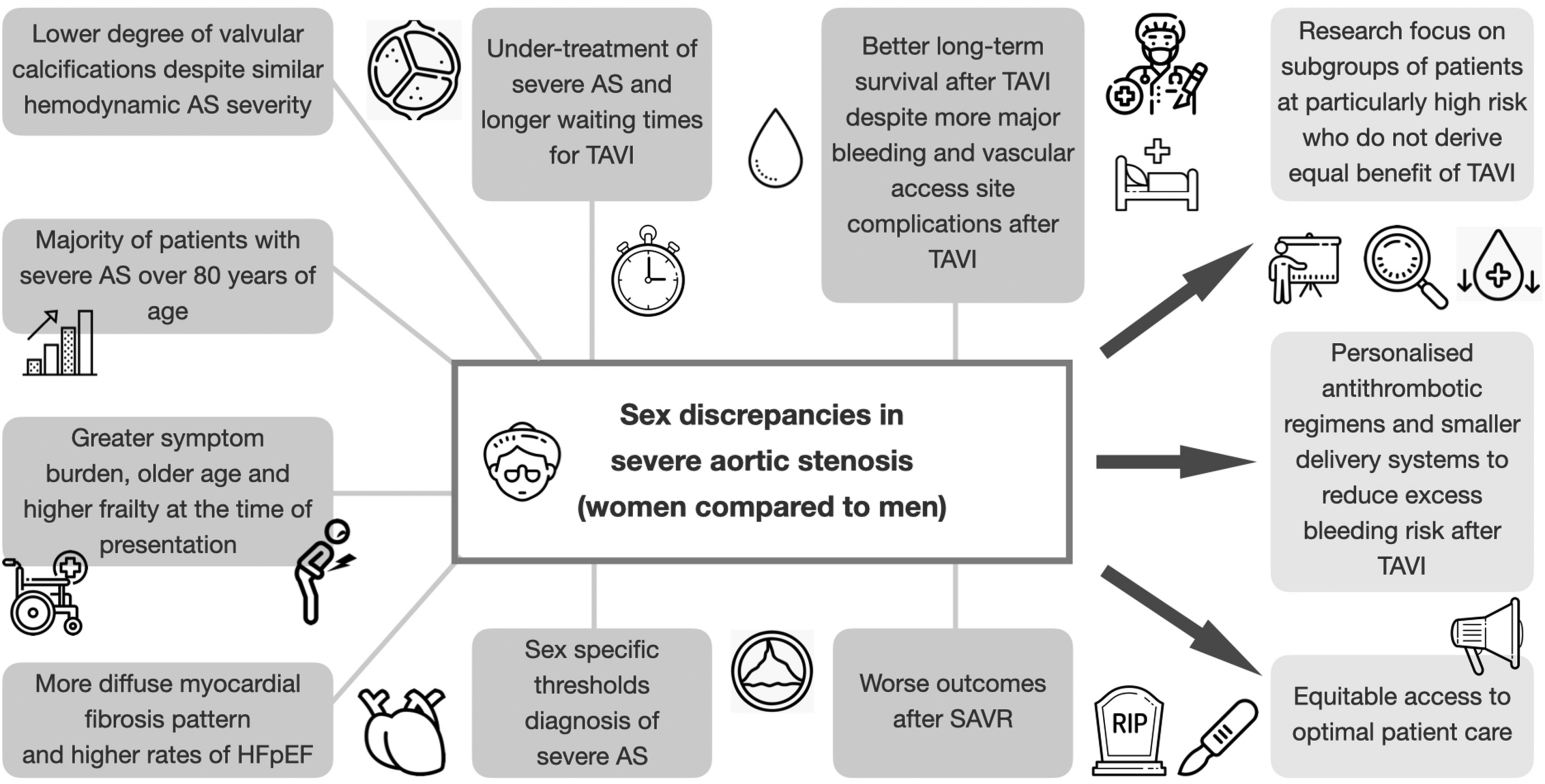
Sex-related discrepancies and future directions in severe aortic stenosis. AS, aortic stenosis; HFpEF, heart failure with preserved ejection fraction; SAVR, surgical aortic valve replacement; TAVI, transcatheter aortic valve implantation.

As discussed above, outcomes after TAVI seem to exhibit less mortality differences between women and men, suggesting that TAVI may mitigate some of the sex-specific disparities observed in SAVR.

### Summary and future directions

In conclusion, sex-related differences are evident from the pathophysiology of AS to clinical presentation, treatment, and outcomes (Central illustration). Aortic stenosis in women is characterized by a lower degree of valvular calcifications and a more diffuse myocardial fibrosis pattern. TAVI has been proven to be an effective treatment option for women with severe AS, contributing to improved survival rates in the mid- to long-term, despite an increased risk of bleeding and procedural complications. However, not all subsets of women seem to derive equal benefit from TAVI. Research should focus on these specific subgroups of patients at particularly high risk, including those with low-gradient, low-ejection fraction AS or those with higher levels of frailty.

To improve outcomes of patients with severe AS, sex specific thresholds for earlier and improved AS diagnosis in women are necessary. Future research is warranted to advance our understanding of sex-specific AV calcification and left ventricular remodeling processes, as well as to develop personalized antithrombotic regimens aiming at reducing the excess bleeding risk of women. Differences in patient referral need to be investigated to ensure equitable access to optimal patient care for women and men with severe AS. Thereby, personalized treatment strategies for patients with severe AS can be developed and outcomes improved.
